# Nanostructure and Corrosion Resistance of Plasma-Based Low-Energy Nitrogen Ion Implanted 17-4PH Martensitic Stainless Steel

**DOI:** 10.3390/nano16030215

**Published:** 2026-02-06

**Authors:** Xu Yang, Honglong Che, Shuyuan Li, Mingkai Lei

**Affiliations:** Surface Engineering Laboratory, School of Materials Science and Engineering, Dalian University of Technology, Dalian 116024, China; yx0727@mail.dlut.edu.cn (X.Y.); chehl@dlut.edu.cn (H.C.); syli@mail.dlut.edu.cn (S.L.)

**Keywords:** 17-4PH martensitic stainless steel, general corrosion resistance, nanocrystalline, plasma-based low-energy nitrogen ion implantation, microstructure

## Abstract

This study aims to enhance the corrosion property of 17-4PH martensitic stainless steel, a material commonly used in industrial applications including nuclear power components, to enhance its performance in borate buffer solutions. The study employed plasma-based low-energy nitrogen ion implantation at temperatures ranging from 350 °C to 550 °C for 4 h to modify the steel surface. Microstructural characterization via XRD and TEM revealed the formation of a nanocrystalline nitrided layer, with thickness increasing from 11 to 27 μm and surface nitrogen concentration rising from 29.7 to 33.1% as temperature increased. Correspondingly, the nanocrystalline grains coarsened from an average size of 2 nm to 15 nm. The main findings showed that all nitrided layers significantly improved general corrosion resistance in pH 8.4 borate solution compared to the unmodified steel. An optimal performance with a corrosion potential of −169.4 mV(SCE) and a passive current density of 0.5 μA/cm^2^ was achieved at 450 °C, accompanying the development of a denser passive film with high polarization resistance and lower defect density. It is concluded that the high interstitial nitrogen concentration within the nanocrystalline γ′_N_ accelerates passivation kinetics and enhances corrosion resistance, with the applied point defect model clarifying the underlying improvement mechanism.

## 1. Introduction

17-4PH martensitic stainless steel is a kind of precipitation-hardened steel featuring martensitic laths and containing fine precipitates enriched in copper or niobium. Owing to the superior strength, ductility and well corrosion resistance, this steel is widely employed for critical structural components of various industrials, including nuclear power, aerospace and petrochemical industries [1,2]. The corrosion resistance property of 17-4PH steel requires further improvement to meet the long-term operational demands under the extended service conditions, such as in nuclear power environments [3].

Plasma-enhanced thermochemical treatment serves as an effective surface modification technique for simultaneously enhancing the corrosion and mechanical properties of 17-4PH steel, including nitriding, carburizing and nitrocarburizing processes [4,5,6]. At low temperatures, below 420 °C, a single expanded martensite α′_N_ phase emerged in the modified layer [7]. The N-supersaturation in the martensitic lattice significantly increased the surface hardness [8]. At high temperature above 420 °C, a mixed phase content of the γ′-Fe_4_N-like ordered nitrogen-expanded austenite γ′_N_, hexagonal close-packed nitride ε-Fe_3_N and CrN precipitates emerged, leading to a further increase in the surface hardness [9]. Wang et al. [10] found that the wear resistance of the nitrided 17-4PH martensitic stainless steel at 350–450 °C was enhanced, leading to a 70–90% reduction in wear loss. Moreover, Riazi et al. [11] reported an increased fatigue strength by 33% for the nitrided layer at 500 °C. In addition to the phase constituents at various temperatures, the grain size also affects the mechanical properties of modified 17-4PH steel. Liu et al. [12] found that the nanocrystalline γ′-Fe_4_N, α′_N_ and CrN with grain sizes of 5–200 nm by plasma nitrocarburizing at 400–500 °C. The nanostructural modified layer with the mixed phases showed a significant increase in surface hardness by 1.5 times and a substantial decrease in wear rate by two orders of magnitude. In pressurized water reactor nuclear power environments, 17-4PH steel commonly experiences general corrosion within borate solutions, a primary failure mechanism initiated by the breakdown and dissolution of passive film [3]. Improving the general corrosion resistance of 17-4PH steel under these conditions is crucial for securing the long-term reliability of key components, such as hydraulic parts. However, the general corrosion behavior in borate aqueous solution for the modified 17-4PH steel has received little attention.

In this article, plasma-based low-energy nitrogen ion implantation (PBLEII) was employed for modifying 17-4PH martensitic stainless steel at 350–550 °C for 4 h to enhance general corrosion resistance property. The microstructure was examined via X-ray diffraction (XRD) and transmission electron microscopy (TEM). General corrosion resistance was assessed in the pH 8.4 borate buffer aqueous solution employing the potentiodynamic polarization curve, electrochemical impedance spectroscopy (EIS), and the Mott–Schottky curve. A point defect model was applied to elucidate the impact of microstructural changes in nitrided 17-4PH steel on the corrosion resistance.

## 2. Materials and Methods

The nominal chemical composition of the commercial 17-4PH martensitic stainless steel is listed in Table 1. A two-stage heat treatment was applied to the material, including the first solution treatment at 1040 °C for 2 h, and subsequently oil-quenching. This was followed by an annealing treatment at 595 °C for 4 h. Cylindrical specimens with a diameter of 17 mm and a thickness of 5 mm were sectioned from the heat-treated stock. Before nitriding, specimens were mechanically ground with successive grades of silicon carbide abrasive papers in 180–2000 grit, polished with 1.5 μm diamond paste, cleaned ultrasonically with acetone for 600 s, and finally dried in air.

Plasma nitriding was performed by a PBLEII system equipped with a high-density, high-ionization-degree electron cyclotron resonance (ECR) microwave plasma source [13]. Before nitriding, the chamber was pumped down to 1.5 × 10^−3^ Pa, followed by introducing nitrogen gas to a constant working pressure of 5 × 10^−2^ Pa throughout the treatment. The −2 kV pulsed negative bias voltage with repetition rate of 1000 Hz and pulse width of 250 μs was employed for the specimen holder. Plasma was sustained with a microwave power of 200 W. Nitriding was carried out at 350, 400, 450, 500, and 550 °C for 4 h. The nitriding temperature was controlled using an auxiliary heater integrated into the specimen holder and monitored in situ by a thermocouple. The average nitrogen ion current density was maintained at 0.8 mA/cm^−2^ to ensure a high implantation dose, with a corresponding plasma density in the range of 5 × 10^11^ to 1.5 × 10^12^ cm^−3^ [13]. After completing the nitriding treatment, the specimens underwent a furnace-cooling process to gradually reduce the temperature. Throughout this stage, a continuous flow of high-purity nitrogen gas was maintained at a constant rate to effectively prevent oxidation and surface degradation. This approach ensured the integrity of the nitrided layer.

For microstructural characterization, cross-sectional specimens were prepared by sequential grinding, polishing, and etching. Etching was carried out by a solution with 10 g FeCl_3_, 10 mL HCl in analytical grade, and 120 mL deionized water. The cross-sectional morphology of the nitrided layer were examined by light-optical microscopy using (LOM, DMi8, Leica Microsystems, DEU, Wetzlar, Germany). Nitrogen concentration-depth profiles were acquired via field-emission electron probe microanalysis (EPMA, JXA-8530F PLUS, JEOL Ltd., Tokyo, Japan) at 30 kV. The ZAF correction was employed for quantitative analysis. Surface nitrogen concentration was determined at three distinct regions on each sample surface, and the average value was used for subsequent analysis. Phase identification was performed by X-ray diffraction (XRD, Empyrean, Malvern Panalytical Ltd., NED, Malvern, UK) using PANalytical Empyrean by Cu Kα radiation with the λ of 0.15405 nm over 20–100°. Further microstructural characterization was conducted with a Field Emission Transmission Electron Microscope (JEM-F200, JEOL Ltd., Tokyo, Japan) at 200 kV, employing dark-field (DF) imaging and selected-area electron diffraction (SAED). TEM foils were prepared from the near-surface region using a Dual-Beam-Focused Ion Beam system (Helios G4 UX, FEI Company, Hillsboro, OR, USA) with Ga^+^ ions. Before milling, a platinum layer was deposited to minimize Ga^+^ induced damage to the nitrided layer.

Electrochemical evaluations were performed on nitrided surfaces with an exposed area of 1 cm^2^ immersed in a pH 8.4 borate buffer aqueous solution. The electrolyte was prepared by dissolving 8.375 g H_3_BO_3_ and 14.3 g Na_2_B_4_O_7_·10H_2_O in 1000 mL deionized water. A conventional three-electrode configuration was used in an EG&G PAR 2273 electrochemical workstation, with the specimen as the working electrode, a platinum mesh as the counter electrode, and a saturated calomel electrode (SCE) as the reference. The corrosive potentials are relative to SCE. Before electrochemical tests, the specimens were polarized cathodically at −800 mV(SCE) for 180 s to remove the oxide film formed in air. Potentiodynamic polarization scanning were initiated from −250 mV relative to open-circuit potential (OCP) with a scanning rate of 0.6 mV·s^−1^. EIS and Mott–Schottky analysis were utilized to assess the impedance and semiconductor characteristics of passive film. For these tests, specimens were immersed at OCP for 1 h to form a stable passive film. A frequency range of 10^−2^ to 10^5^ Hz was selected for the EIS with a 10 mV sinusoidal perturbation. Data were fitted using ZSimpWin software (version 3.30d). Mott–Schottky plots were recorded from −2000 to 1000 mV(SCE) with a 10 mV AC amplitude at 1000 Hz and a DC potential scan rate of 20 mV·s^−1^. The electrochemical tests were conducted three times to ensure reproducibility.

## 3. Results

### 3.1. Microstructure of the Nitrided Layer

**Figure 1:**
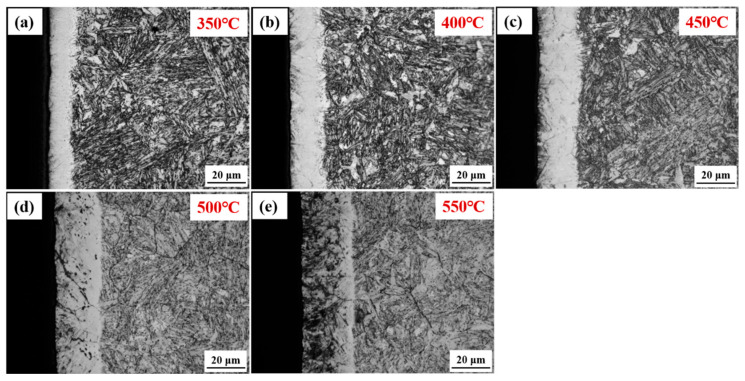
Optical micrographs of the cross-sectional nitrided 17-4PH steel at (**a**) 350 °C; (**b**) 400 °C; (**c**) 450 °C; (**d**) 500 °C; and (**e**) 550 °C.

Figure 1 shows the optical micrographs of the cross-sectional nitrided 17-4PH steel at 350–550 °C for 4 h. The matrix underlying the nitrided layers exhibits a microstructure consistent with that of lath martensite (α’) and a minor fraction of residual austenite (γ_re_). At 350 °C, a continuous white layer with an average thickness of approximately 11 μm forms on the steel surface. The average layer thickness increases with nitriding temperature, measuring approximately 21 μm at 450 °C and further increasing to approximately 27 μm at 550 °C. Furthermore, precipitates are first observed along grain boundaries at 400 °C. The depth of these precipitates increases progressively with nitriding temperature from 400 °C to 550 °C, as illustrated in Figure 1b–e.

**Figure 2:**
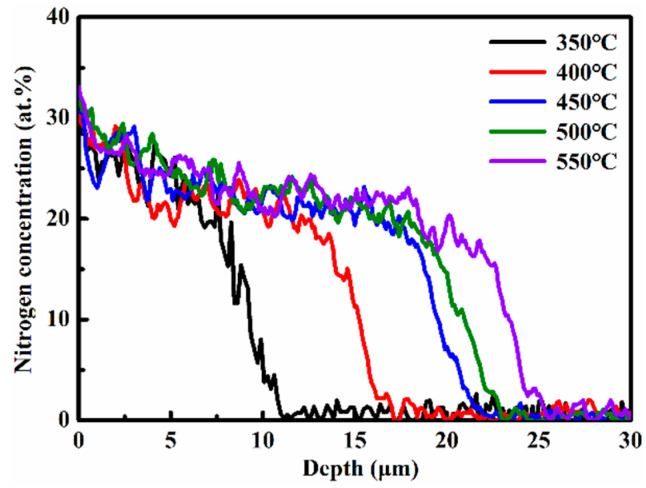
Nitrogen concentration-depth profiles of the nitrided 17-4PH steel at 350–550 °C for 4 h.

Figure 2 shows the nitrogen concentration-depth profiles of the nitrided 17-4PH steel at 350–550 °C for 4 h. At the lowest temperature of 350 °C, the surface nitrogen concentration reaches a maximum of approximately 29.7%. This concentration increases steadily with rising nitriding temperature, reaching about 30.6% at 450 °C and further rising to 33.1% at 550 °C. The observed enhancement in surface nitrogen content with temperature is consistent with accelerated inward diffusion of nitrogen at higher processing temperatures.

All nitrogen concentration-depth profiles exhibit a high concentration plateau with a steep gradient profile across the transition zone between the nitrided layer and substrate. This nitrogen concentration distribution is attributed to trapping–detrapping effect of interstitial nitrogen atoms with chromium atoms in solid solution [14]. With the nitriding temperature range of 350–550 °C, the nitrogen penetration depths in the nitrided layers vary from approximately 11 μm to 26 μm.

**Figure 3:**
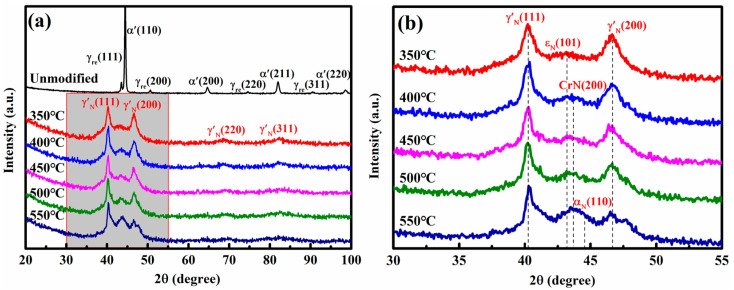
XRD patterns of the unmodified and nitrided 17-4PH steel at 350–550 °C, (**a**) the full diffraction pattern, (**b**) the magnified diffraction pattern correspond to the grey area in (**a**).

Figure 3 shows the XRD patterns of the unmodified and nitrided 17-4PH steel at 350–550 °C for 4 h. The diffraction peaks of martensite (α’) and retained austenite (γ_re_) are observed for the unmodified steel. After nitriding, all diffractograms exhibit peaks corresponding to nitrogen-expanded austenite with a γ′-Fe_4_N-type structure (denoted as γ’_N_) and the hexagonal close-packed (HCP) ε_N_ phase. Compared with the γ_re_ reflections, the γ’_N_ peaks are shifted toward lower 2θ angles due to the lattice expansion induced by the interstitial dissolution of nitrogen in the face-centered cubic (FCC) matrix. The observed peak broadening primarily results from grain refinement or high density of defects [15]. As the nitriding temperature rises, these peaks become sharper, indicating coarsening of the grains. Moreover, the ε_N_ with a diffraction peak of approximately 43.2° is considered a stacking fault structure in the nitrided layers [16]. A diffraction peak near 43.5° can be assigned to CrN. Its intensity increases with nitriding temperature, reflecting a higher volume fraction of CrN precipitates, as consistent with the observation in Figure 1e. At 550 °C, an additional peak associated with nitrogen-containing ferrite α_N_ emerges, suggesting the onset of γ’_N_ decomposition.

**Figure 4:**
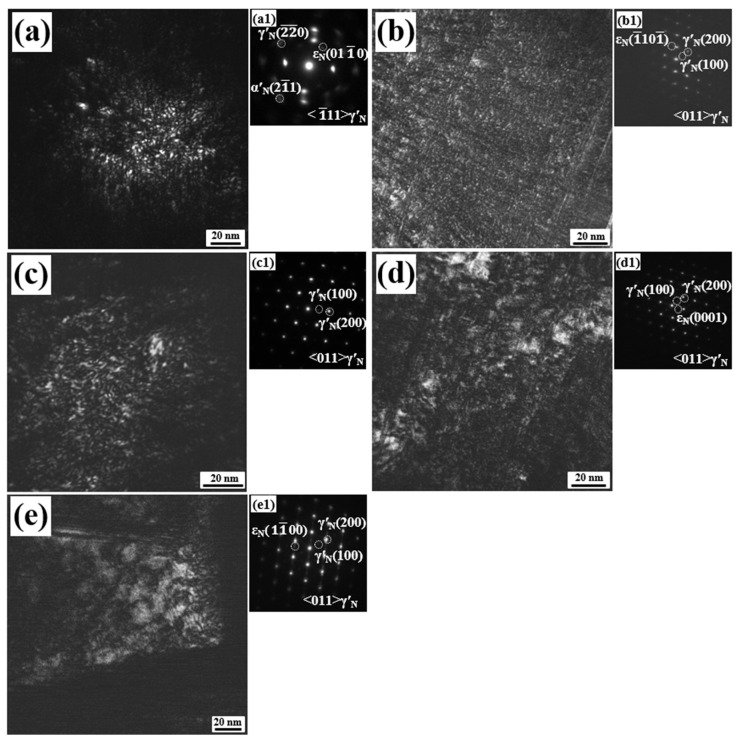
TEM results of the nitrided 17-4PH steel. (**a**–**e**) DF-TEM images for the nitrided layers at 350–550 °C for 4 h. (**a1**–**e1**) The corresponding SAED patterns.

Figure 4 shows the DF-TEM images and the corresponding SAED patterns of nitrided 17-4PH steel at 350–550 °C for 4 h. The SAED patterns in Figure 4(a1–e1) correspond directly to the respective DF-TEM micrographs. Across the entire studied temperature range, the nitrided layer consistently exhibited a nanocrystalline structure, confirming that low-temperature plasma nitriding induces nanocrystallization in this alloy. The nanocrystallization is driven by the coupling of chemical and elastic fields at low temperature. The coupling effect triggers a self-sustaining periodic diffusionless austenitic transformation in nanoscale. This transformation process results in sequential layer-by-layer nanocrystallization, as introduced in another work more detail [17]. The average grain size increased gradually with nitriding temperature, from approximately 2 nm at 350 °C to roughly 15 nm at 550 °C, because of the higher mobility for metal atoms. This trend agrees well with the XRD results presented earlier.

### 3.2. Corrosion Properties of the Nitrided Layer

#### 3.2.1. Polarization Characteristics

**Figure 5:**
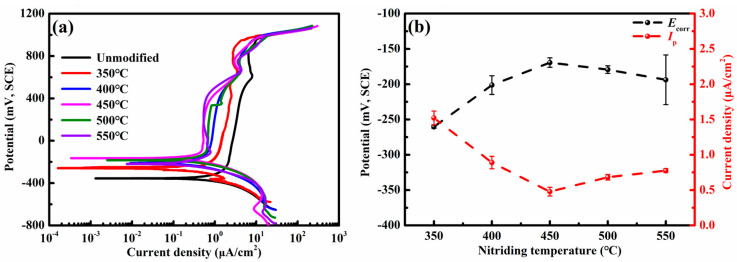
Polarization curves of the unmodified and nitrided 17-4PH steel in the borate buffer solution. (**a**) Potentiodynamic polarization curves; (**b**) corrosion potential *E*_corr_ and passivation current densities *I*_p_ at temperature from 350–550 °C, and the dashed line indicates the variation tendency.

Figure 5a shows the potentiodynamic polarization curves of the unmodified and nitrided 17-4PH steel in the pH 8.4 borate buffer aqueous solution. Both the unmodified and nitrided specimens display a similar active-to-passive transition, followed by transpassive dissolution. The corrosion potential (*E*_corr_) and passivation current density (*I*_p_) are extracted and summarized in Figure 5b. Relative to the unmodified specimen with *E*_corr_ of −371.6 mV(SCE) and *I*_p_ of 2.3 μA·cm^−2^, the nitrided layers exhibit nobler *E*_corr_ and lower *I*_p_ values, indicating improved thermodynamic stability and more readily activated passivation characteristics. Increasing the nitriding temperature from 350 °C to 450 °C, the *E*_corr_ shifts positively, while the *I*_p_ decreases, and the trend reverses beyond 450 °C. Consequently, the general corrosion resistance is enhanced at nitriding temperatures between 350 °C and 450 °C, but deteriorates when the temperature reaches 550 °C. The optimal performance is achieved after nitriding at 450 °C for 4 h, with a *E*_corr_ of −169.4 mV(SCE) and a *I*_p_ of 0.5 μA·cm^−2^. Notably, the nitrided AISI 304L austenitic stainless steel under similar nitriding conditions (400 °C, 4 h) exhibits a *E*_corr_ of −231.0 mV(SCE) and a *I*_p_ of 1–3 μA·cm^−2^ [18]. The nitrided 17-4PH steel at 450 °C exhibits a positive shift in *E*_corr_ of about 61.6 mV and an order-of-magnitude lower *I*_p_, demonstrating significantly superior general corrosion resistance in the same corrosive condition.

#### 3.2.2. Impedance Characteristics

**Figure 6:**
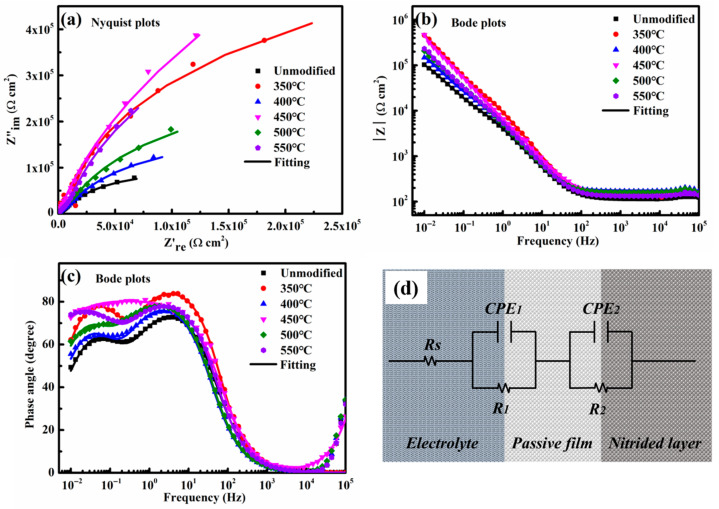
Impedance characteristics of the unmodified and nitrided 17-4PH steel immersed in the borate buffer aqueous solution at the OCP for 1 h. (**a**) Nyquist plots; (**b**,**c**) Bode plots; and (**d**) equivalent electrical circuit diagram.

Figure 6 shows the EIS plots and the corresponding equivalent circuit model for the unmodified and nitrided 17-4PH steel immersed in the borate buffer aqueous solution at the OCP for 1 h. Within the measured frequency range, all Nyquist plots display a single capacitive arc. A larger arc radius generally suggests the superior corrosion resistance [19]. Notably, the nitrided 17-4PH steel at 350–550 °C exhibit substantially larger arc radii than the unmodified steel, confirming the improved general corrosion resistance of 17-4PH martensitic stainless steel by plasma nitriding. Among the processed specimens, the layer nitrided at 450 °C demonstrates the highest corrosion resistance, as reflected by the largest capacitive arc in the Nyquist plot. The Bode magnitude plots further support these observations. In the high-frequency region, the impedance modulus (|Z|) exhibits a plateau, while in the low-frequency region, all nitrided specimens show higher |Z| values than the untreated steel. Specifically, at a low frequency of 10^−2^ Hz, the nitrided layers prepared at 350 °C and 450 °C both reach a maximum |Z| value of approximately 4.70 × 10^5^ Ω·cm^2^.

The nitrided 17-4PH steels at 350–550 °C exhibit a similar bimodal distribution in phase angle across the frequency range of 10^−2^–10^2^ Hz, except for the nitrided steel at 450 °C. At 450 °C, a well-defined plateau region is presented for the phase angle within the frequency range of 10^−2^–10^1^ Hz, as shown in Figure 6c. The nitrided 17-4PH steels at 350–550 °C exhibit a higher phase angle compared with the unmodified steel, indicating the improved passive film stability in the pH 8.4 borate buffer solution. Within the frequency range of 10^0^–10^1^ Hz, a highest phase angle of 83.7° is recorded at 350 °C. Notably, the nitrided steel at 450 °C maintains a high phase angle of 80.3° over an extended plateau frequency range of 10^−2^–10^1^ Hz, suggesting high charge transfer resistance and suppressed corrosion kinetics. A more stable passive film forms on the nitrided 17-4PH steel with the improved general corrosion resistance in the pH 8.4 borate buffer aqueous solution.

The EIS data obtained for both unmodified and nitrided 17-4PH steel in the pH 8.4 borate buffer aqueous solution were fitted by an equivalent electrical circuit with two-time constants in Figure 6d. The impedance characteristics of a passive film directly correspond to its compactness and stability. In this model, the capacitive behavior is represented by the constant-phase element (CPE), which accounts for the non-ideal capacitance typically observed in stainless-steel systems [20]. The impedance of CPE is given via the following expression [21]:

*Z*_CPE_ = *Y*_0_^−1^(*jω*)^−*n*^ (1)

where *Y*_0_ is the admittance value, *ω* represents the angular frequency, *j* is the imaginary number, and *n* is the dispersion coefficient. When the coefficient *n* = 1, the CPE represents a pure capacitance, while when *n* < 1, the *CPE* exhibits a non-ideal capacitance. In this circuit, *R*_s_ denotes the solution resistance, *R*_1_ and *R*_2_ represent the passive film resistance and the charge transfer resistance, respectively, *CPE*_1_ and *CPE*_2_ correspond to the capacitances of passive film and double electric layer. The total ohmic resistance of the equivalent electrical circuit is represented by polarization resistance (*R*_p_), defined as *R*_p_ = *R*_1_ + *R*_2_ [22]. The fitted electrochemical parameters and the calculated *R*_p_ values are summarized in Table 2.

In the pH 8.4 borate buffer solution, the nitrided steel exhibits significantly higher polarization resistance *R*_p_ in the temperature range of 350–550 °C compared to the unmodified stainless steel (2.00 × 10^5^ Ω·cm^2^). The improved resistance is caused by the development of a more stable passive film. Increasing the nitriding temperature from 350 °C to 450 °C, *R*_p_ rises from 2.13 × 10^5^ Ω·cm^2^ to a maximum of 4.68 × 10^5^ Ω·cm^2^. Further increasing the temperature to 550 °C, however, leads to a decline in *R*_p_ to 2.54 × 10^5^ Ω·cm^2^. The sample nitrided at 450 °C demonstrates the highest *R*_p_ value, indicating its optimal general corrosion resistance in the tested environment.

#### 3.2.3. Semiconductor Characteristics

The corrosion resistance of stainless steel is inherently linked to the semiconductor characteristics of its passive film [23]. Mott–Schottky analysis, which presents the relation between the inverse square of space charge capacitance (*C*^−2^) and potential (*E*), provides insight into the electronic properties of this film. The donor density (*N*_D_) and acceptor density (*N*_A_) can be quantitatively determined from the linear regions of the Mott–Schottky plot using the following relationships:

(2) $$\frac{1}{C^{2}}\;=\;\frac{2}{\varepsilon \varepsilon_{0}eN_{D}}(E\;-E_{\mathrm{fb}}-\frac{kT}{e})\;\mathrm{for}\;n\text{-}\mathrm{type}\;\mathrm{semiconductor}$$

(3) $$\frac{1}{C^{2}}=\frac{-2}{\varepsilon \varepsilon_{0}eN_{A}}(E-E_{\mathrm{fb}}-\frac{kT}{e})\;\mathrm{for}\;p\text{-}\mathrm{type}\;\mathrm{semiconductor}$$

where *ε* = 15.60 F/m represents the dielectric constant of passive film composed mainly of Cr(OH)_3_/(Fe_2_O_3_ + Cr_2_O_3_), *ε*_0_ = 8.85 × 10^−14^ F/cm is the vacuum permittivity, *e* = 1.60 × 10^−19^ C denotes the electron charge, *N* corresponds to the charge carrier density, including *N*_D_ and *N*_A_, *E*_fb_ is the flat-band potential, *k* = 1.38 × 10^−23^ J/K is the Boltzmann constant, and *T* is the absolute temperature [24]. A positive slope in the *C*^−2^ vs. *E* plot indicates *n*-type semiconductor behavior, while a negative slope suggests *p*-type semiconductor behavior. The charge carrier density is inversely proportional to the slope magnitude according to the given relationship.

(4) $$N\;=\frac{2}{\varepsilon \varepsilon_{0}eS}$$

where *N* is the charge carrier densities, *S* represents the absolute value of the slope in the linear region of plots. The flat-band potential (*E*_fb_) is obtained by the following equation:

(5) $$E_{\mathrm{fb}}\;=\;E\;-\frac{\varepsilon \varepsilon_{0}eN_{D}}{2C^{2}}\;-\;\frac{kT}{e}$$

**Figure 7:**
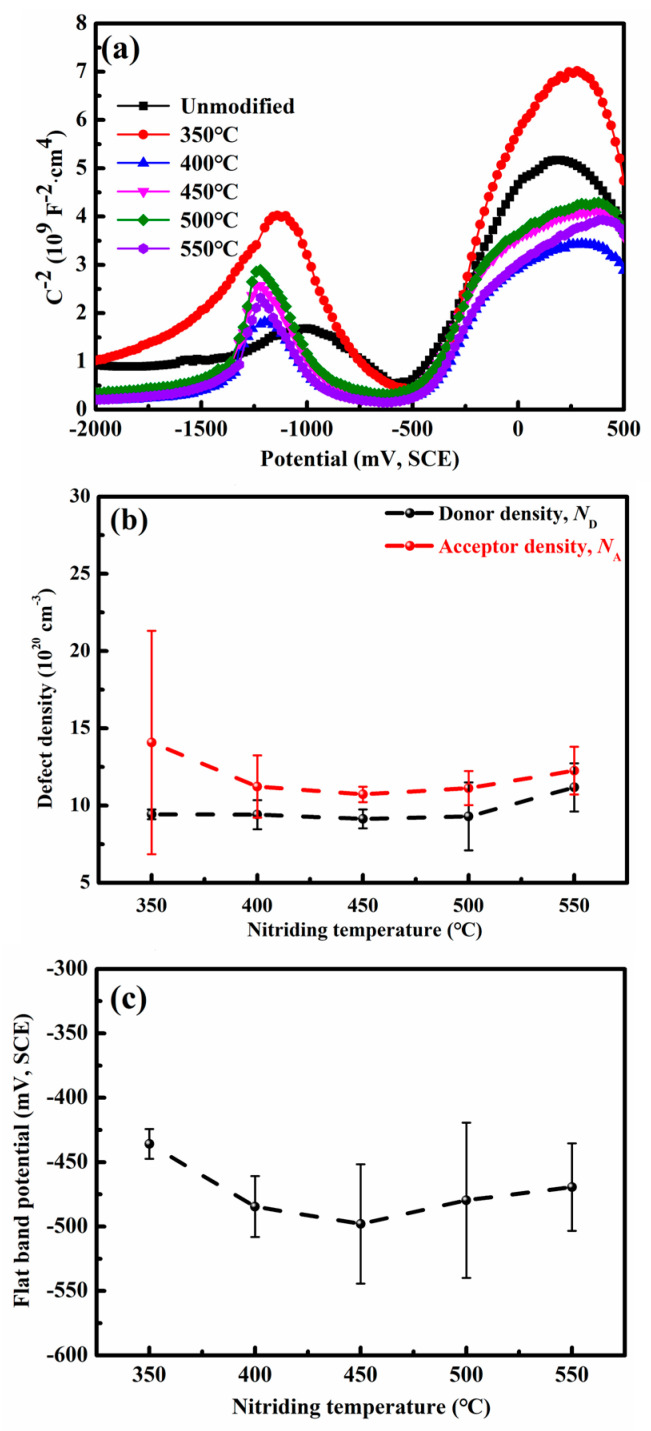
Semiconductor characteristics of the passive film for the nitrided 17-4PH steel immersed in the pH 8.4 borate buffer aqueous solution at the OCP for 1 h. (**a**) Mott–Schottky curves; (**b**) carrier densities; and (**c**) flat-band potentials.

Mott–Schottky analysis was utilized to examine the electronic characteristics of passive films on nitrided 17-4PH steel. Figure 7 shows the Mott–Schottky plots, charge carrier densities, and flat-band potentials for the unmodified and nitrided 17-4PH steel at 350–550 °C for 4 h. Measurements were conducted in the pH 8.4 borate buffer aqueous solution at the OCP for 1 h. The Mott–Schottky response of the nitrided stainless steels is qualitatively similar to that of the unmodified steel, suggesting a comparable electronic structure in their respective passive films. The passive film on 17-4PH steel exhibits characteristic potential-dependent behavior. Three potential regions are determined based on the reaction behaviors at the stainless steel–passive film and passive film–solution interfaces. For the unmodified steel, the potential range of −1060 mV(SCE) to −780 mV(SCE) corresponds to *p*-type semiconductor behavior with a negative slope, denoted as Region III. A potential-independent capacitance plateau with flat-band condition is presented in the potential range of −780 mV(SCE) to −360 mV(SCE), denoted as Region II. The potential range of −360 mV(SCE) to 20 mV(SCE) corresponds to *n*-type semiconductor behavior with a positive slope in Region I. For the nitrided steel at 350 °C, Region III ranges from −880 mV(SCE) to −640 mV(SCE), Region II spans from −640 mV(SCE) to −400 mV(SCE) and Region I defines as −400 mV(SCE) to −60 mV(SCE). For the nitrided steel at 400–550 °C, Region III corresponds to −1140 mV(SCE) to −900 mV(SCE), Region II spans from −900 mV(SCE) to −400 mV(SCE) and Region I covers the potential range of −400 mV(SCE) to −160 mV(SCE).

The corrosion resistance of stainless steels is intrinsically associated with the passive film quality, which is influenced by defect density. In this work, the *N*_D_ and *N*_A_ for the nitrided stainless steel at 350–550 °C were evaluated via Mott–Schottky analysis. As summarized in Figure 7b, both *N*_D_ and *N*_A_ for all nitrided specimens are lower than those of the unmodified stainless steel with 11.64 × 10^20^ cm^−3^ and 23.16 × 10^20^ cm^−3^, respectively, indicating enhanced general corrosion resistance. Notably, the reduction in *N*_D_ is less pronounced than that in *N*_A_. With increasing nitriding temperature from 350 °C to 450 °C, *N*_A_ decreases from 14.08 × 10^20^ cm^−3^ to 10.72 × 10^20^ cm^−3^, then rises slightly to 12.26 × 10^20^ cm^−3^ at 550 °C. This non-monotonic trend suggests the development of a denser protective passive film at 450 °C. Similarly, *N*_D_ follows a comparable trend across the same temperature range, reaching its minimum value of 9.14 × 10^20^ cm^−3^ at 450 °C. These results collectively demonstrate that nitriding at 450 °C yields the most defect-resistant passive film, thereby optimizing corrosion performance.

The *E*_fb_ for the nitrided 17-4PH steel at 350–550 °C are consistently lower than that for the unmodified steel, which exhibits an *E*_fb_ of −396.5 mV(SCE) in Figure 7c. Among these, the nitrided steel at 450 °C shows the most negative *E*_fb_ value of −484.5 mV(SCE). In semiconductor electrochemistry, *E*_fb_ is inversely related to the Fermi level (*E*_f_). A more negative *E*_fb_ indicates a lower density of charge carriers capable of overcoming the interfacial energy barrier, thereby impeding charge transfer across the film. Consequently, a passive film with superior protective properties forms on the nitrided steel at 450 °C.

## 4. Discussion

### 4.1. Microstructural Evolution

**Figure 8:**
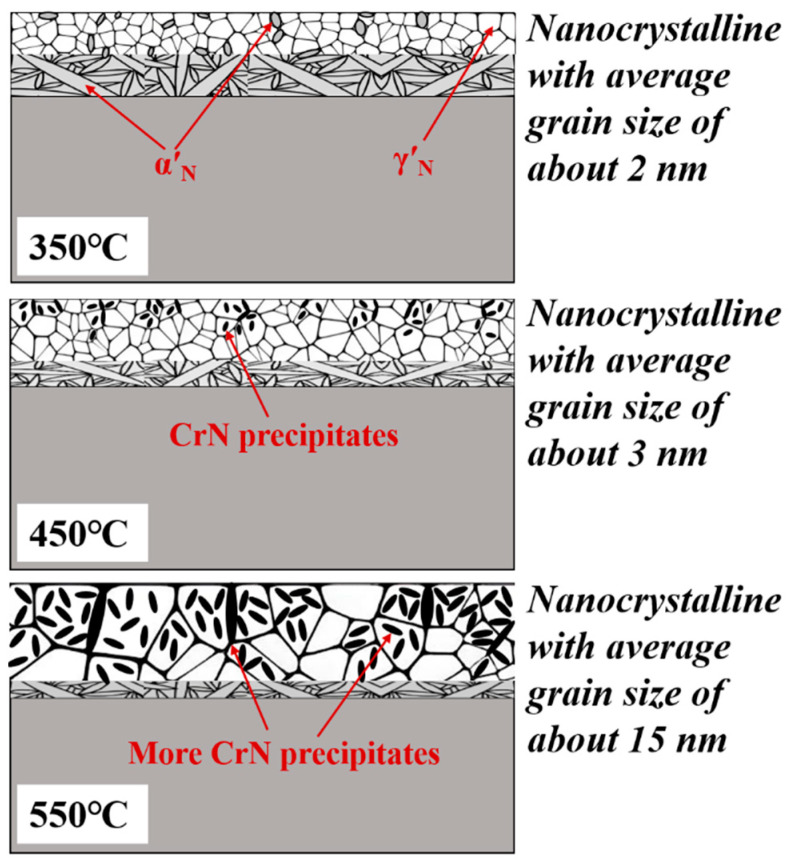
Schematic diagrams of the microstructural evolution on the nitrided 17-4PH steel at 350–550 °C for 4 h.

Figure 8 shows the microstructural evolution of the nitrided layer within the 350–550 °C temperature range. The nitrided layer exhibits a dispersed distribution of nanocrystals with an average grain size of 2–15 nm. Our previous research has confirmed that low temperature nitriding of martensitic stainless steel represents the non-equilibrium kinetic process driven by the coupling of two diffusion-controlled fields of chemical and elastic. At the diffusion front, the cyclic accumulation of chemical and elastic energy reaches a critical threshold, triggering a diffusionless austenitic transformation. The released energy and continued diffusion create a self-sustaining periodic mechanism, resulting in sequential layer-by-layer transformation and nanocrystallization [17]. As the nitriding temperature increases, the long-range movement of metal atoms intensifies, not only promoting the coarsening of nanostructures, but also leading to the development of more CrN.

### 4.2. Corrosion Mechanism

The corrosion resistance of nitrided 17-4PH steel is determined by its phase composition and nanocrystalline structure [3,25]. In borate buffer solution, the layers nitrided at 350–550 °C exhibit a lower corrosion rate relative to the unmodified steel. EIS and Mott–Schottky analysis confirm that a more protective passive film forms on the nitrided surfaces. This enhanced general corrosion resistance originates from the synergistic effects of interstitial nitrogen atoms within the steel matrix, coupled with the nanocrystalline structure. Interstitial nitrogen neutralizes H^+^ ions in the solution, raising the local pH. This pH shift decelerates passive film dissolution and accelerates repassivation kinetics [26]. Furthermore, nitrogen accumulation reduces defect density within the passive film by occupying anion vacancies [27]. Additionally, the nanocrystalline microstructure with high grain boundary density provides extensive pathways for the inward diffusion of oxygen atoms and the outward migration of metal atoms [28]. This process promotes the rapid development of a dense passive film with oxides and hydroxides.

The general corrosion resistance of nitrided 17-4PH steel in the pH 8.4 borate buffer aqueous solution improves as the nitriding temperature increases from 350 °C to 450 °C, but deteriorates upon further heating to 550 °C. At the lower nitriding temperatures of 350 °C to 450 °C, the increasing concentration of interstitial nitrogen within the nanocrystalline γ′_N_ phase accelerates passivation and promotes a denser passive film. Although minor CrN precipitation occurs at 400–450 °C, its detrimental impact on corrosion resistance is mitigated by the high interstitial nitrogen content [29]. In contrast, at 550 °C, extensive decomposition of γ′_N_ leads to an increase in the volume fraction of CrN. Excessive CrN precipitation depletes both interstitial nitrogen and chromium from the solid solution [30,31], impairing the protective quality of the passive film. Simultaneously, the coarsening of the nanocrystalline structure reduces the density of fast-diffusion pathways for oxygen and metal atoms [32]. Consequently, a thinner and less effective passive film forms at higher nitriding temperatures, resulting in degraded corrosion resistance at 500–550 °C.

Overall, the general corrosion resistance of the nitrided 17-4PH steel is associated with two effects, including the beneficial effect of interstitial nitrogen atoms in the γ′_N_ phase and the detrimental impact of CrN precipitates within the nitrided layer. With increasing nitriding temperature, the surface nitrogen concentration rises progressively, accompanied by a gradual increase in the volume fraction of the CrN. At the nitriding temperatures of 350–450 °C, the beneficial effect of nitrogen atoms dominates the improved general corrosion resistance. In contrast, the detrimental impact of CrN precipitates becomes dominant at temperatures above 450 °C.

### 4.3. Applied Point Defect Model

**Figure 9:**
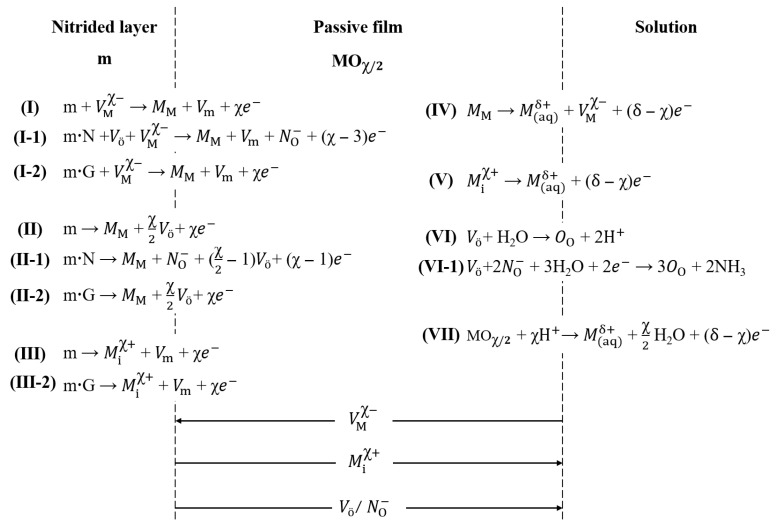
Schematic illustration of reactions at nitrided layer–passive film and passive film–solution interfaces according to the applied PDM. m = metal atoms, $V_{M}^{x-}$ = cation vacancies, $M_{M}$ = metal ions in cation sites, $V_{m}$ = vacancies in metal, m·N = metal atoms together with nitrogen atoms, $N_{O}^{-}$ = nitrogen atoms in anion sites, m·G = metal atoms at grain boundaries, $V_{ö}$ = anion vacancies, $M_{i}^{x+}$ = cation interstitials, $M_{\left(\mathrm{aq}\right)}^{\delta +}$ = metal ions in aqueous solution, $O_{O}$ = oxygen ions in anion sites, and ${\mathrm{MO}}_{x/2}$ = metal oxides, The arrow stands for the moving direction of vacancies or atoms.

To further clarify the influence of phase constituents and nanocrystalline microstructures on the general corrosion behavior, an applied point defect model (PDM) was introduced for the nitrided 17-4 PH steel in borate buffer solution. This model investigates the development and dissolution of passive film via modifying the reactions at the nitrided layer–passive film and passive film–solution interfaces [33], considering the interstitial nitrogen atoms and the nanostructure. Figure 9 shows the schematic illustration of the interfacial reactions according to the applied PDM. The defect categories of passive film primarily consist of acceptor density, i.e., cation vacancies ($V_{M}^{x-}$), and donor density, i.e., anion vacancies ($V_{ö}$) and cation interstitials ($M_{i}^{x+}$) [33,34]. Reactions (I) and (IV) describe the annihilation and generation of the cation vacancies ($V_{M}^{x-}$), respectively. Similarly, reactions (II) and (VI) represent the formation and annihilation of the anion vacancies ($V_{ö}$), respectively. Reactions (III) and (V) depict the formation and annihilation of the cation interstitials ($M_{i}^{x+}$), respectively. Consequently, $V_{M}^{x-}$ migrate from the passive film–solution interface toward the nitrided layer–passive film interface, while $V_{ö}$ and $M_{i}^{x+}$ diffuse in the opposite direction. Besides the defects categories, the passive film (${\mathrm{MO}}_{x/2}$) also consists of metal ions in cation sites ($M_{M}$) and oxygen ions in anion sites ($O_{O}$), as demonstrated by reactions (I), (II), and (VI) [34]. Reactions (II) and (VII) govern the development and dissolution of passive film.

In the nitrided 17-4PH steel, the beneficial effect of interstitial nitrogen atoms primarily originates from the hydrolysis process. During the formation of passive film, nitrogen atoms migrate from the passive film toward the solution via $V_{ö}$ by reaction I-1. These nitrogen atoms neutralize H^+^ ions by reaction (VI-1) and then inhibit the passive film dissolution by reaction (VII) [24,26]. This process elevates the corrosion potential *E*_corr_ of the nitrided steel compared to the unmodified steel, as shown in Figure 5b. The increased consumptions of $V_{M}^{x-}$ and $V_{ö}$ induced by nitrogen atoms in reaction (I-1) result in the low acceptor density *N*_A_ and donor density *N*_D_ in Figure 7b. Reactions (II-1) and (VI-1) revealed that nitrogen atoms promote the passivation of the nitrided layer, characterized by the low passivation current density *I*_p_ in Figure 5b. The electronic conductivity of the passive film is modulated by $V_{M}^{x-}$ and $V_{ö}$. Consequently, the increased consumptions of these vacancies also enhance the electrochemical impedance of passive film, leading to the high polarization resistance *R*_p_ for the nitrided layers.

The nanocrystalline microstructure with high grain boundary density facilitates rapid dissolution of metal atoms due to the reduced electron work function [35]. Thus, the nanostructure modulates the reactions (I-2), (II-2), and (III-2) at the nitrided layer–passive film interface, as shown in Figure 9. Here, m·G represents the metal atoms at grain boundaries. The nanocrystalline microstructure promotes the formation of $M_{M}$ and $V_{ö}$ by reaction (II-2) [34]. The accumulation of $V_{ö}$ accelerates its diffusion to the passive film–solution interface. The formation of $O_{O}$ by reaction (VI) thickens the dense passive film. A significant consumption of $V_{M}^{x-}$ at the nitrided layer–passive film interface decreases the acceptor density *N*_A_ by reaction (I-2). The interstitial nitrogen atoms in the nanocrystalline γ′_N_ migrate to the passive film–solution interface by occupying the $V_{ö}$, as confirmed by the formation of $N_{o}^{-}$ in reactions (I-1) and (II-1). This process reduces the donor density *N*_D_ for the passive film. Concurrently, the nanocrystalline microstructure promotes the development of $M_{i}^{x+}$ by reaction (III-2), characterized by the increased donor density *N*_D_. Owing to the combined effects of the interstitial nitrogen atoms and the nanocrystalline microstructure, a comparatively smaller reduction in donor density *N*_D_ is determined relative to the decrease in acceptor density *N*_A_ shown in Figure 7b.

## 5. Conclusions

(1) PBLEII was employed to modify 17-4PH martensitic stainless steel at 350–550 °C for 4 h. A nanocrystalline microstructure forms on the nitrided surface. As the nitriding temperature increased, the layer thickness increased from 11 μm to 27 μm, and the maximum surface nitrogen concentration rose from 29.7% to 33.1%. Concurrently, the nanocrystalline grain size coarsened from approximately 2 nm to 15 nm.
(2) All nitrided layers exhibit enhanced general corrosion resistance in the pH 8.4 borate buffer aqueous solution compared to the untreated steel. The improvements are primarily attributed to the presence of nanocrystalline γ′_N_, with minor variations due to CrN precipitation. The optimal corrosion performance is achieved in the nitrided 17-4PH steel at 450 °C, which features nanocrystalline γ′_N_ with 3 nm and minor CrN. This layer exhibits a nobler corrosion potential of −169.4 mV(SCE) and a reduced passivation current density of 0.5 μA·cm^−2^.
(3) The point defect model was utilized to elucidate the mechanism underlying the improved corrosion resistance. The high interstitial nitrogen concentration within the nanocrystalline γ′_N_ accelerates passivation kinetics. The nitrided 17-4PH steel at 450 °C forms a particularly dense passive film, characterized by a high polarization resistance (*R*_P_) of 4.68 × 10^5^ Ω·cm^2^ and lower charge carrier densities, acceptor density (*N*_A_) of 10.72 × 10^2^ cm^−3^ and donor density (*N*_D_) of 9.14 × 10^20^ cm^−3^, which correlates with its superior barrier properties.

## Figures and Tables

**Table 1:** Chemical composition of 17-4PH martensitic stainless steel (in wt.%).

C	Si	Mn	Cr	Ni	Cu	Nb	P	S	Fe
≤0.07	0.40	0.82	16.03	4.46	4.25	0.32	≤0.022	≤0.010	bal.

**Table 2:** Electrochemical fitting data for the nitrided 17-4PH steel immersed in the pH 8.4 borate buffer aqueous solution at the OCP for 1 h.

	*R*_s_ (Ω·cm^2^)	*Y*_1_ (Ω^−1^·cm^−2^·s*^n^*)	*n*_1_	*R*_1_ (Ω·cm^2^)	*Y*_2_ (Ω^−1^·cm^−2^·s*^n^*)	*n*_2_	*R*_2_ (Ω·cm^2^)	*R*_p_ (Ω·cm^2^)
Unmodified	119.5	9.09 × 10^−5^	0.886	1.96 × 10^5^	8.25 × 10^−5^	0.928	4.86 × 10^3^	2.00 × 10^5^
350 °C	150.8	3.70 × 10^−5^	0.901	2.11 × 10^5^	3.10 × 10^−5^	0.947	2.04 × 10^3^	2.13 × 10^5^
400 °C	137.0	7.09 × 10^−5^	0895	2.01 × 10^5^	3.01 × 10^−5^	0.931	1.79 × 10^3^	2.03 × 10^5^
450 °C	152.0	2.87 × 10^−5^	0.989	4.60 × 10^5^	4.95 × 10^−5^	0.994	7.51 × 10^3^	4.68 × 10^5^
500 °C	166.1	9.02 × 10^−5^	0.933	2.06 × 10^5^	5.78 × 10^−5^	0.929	4.43 × 10^3^	2.10 × 10^5^
550 °C	137.8	5.41 × 10^−5^	0.934	2.46 × 10^5^	7.74 × 10^−5^	0.941	7.63 × 10^3^	2.54 × 10^5^

## Data Availability

The original contributions presented in this study are included in the article. Further inquiries can be directed to the corresponding author.
